# Diagnosis of Brachiocephalic Thrombophlebitis as the Cause of Fever of Unknown Origin by 18F-FDG-PET/CT

**DOI:** 10.4274/mirt.47966

**Published:** 2015-02-15

**Authors:** Anastas Demirev, Boudewijn Brans, Floris Vanmolkot, Rick De Graaf, Felix Mottaghy, Jan Bucerius

**Affiliations:** 1 Maastricht University Faculty of Medicine, Department of Nuclear Medicine, Maastricht, Netherlands; 2 Maastricht University Faculty of Medicine, Department of Internal Medicine, Maastricht, Netherlands; 3 Maastricht University Faculty of Medicine, Department of Radiology, Maastricht, Netherlands; 4 Rwth Aachen University Faculty of Medicine Hospital, Clinic of Nuclear Medicine, Aachen, Germany; 5 Maastricht University Faculty of Medicine, Medical Cardiovascular Research Institute Maastricht, Netherlands

**Keywords:** Thrombophlebitis, fever of unknown origin, 18F-FDG, Positron-emission tomography

## Abstract

Fever of unknown origin (FUO) represents a challenge in diagnosis and treatment. The role of 18Ffluorodeoxyglucose positron emission tomography (FDG-PET) / computed tomography (CT) in the differential diagnosis of this entity is presently well established. We report the case of a patient with infectious/inflammatory symptoms but no evident localization and subsequent relapse, in which PET/CT showed its ability to not only determine the exact localization of a thrombophlebitic focus as cause of FUO, but also to monitor and determine the success of treatment. After performing a FDG-PET/CT and detecting a thrombophlebitis in the brachiocephalic vein, low molecular heparins were introduced in the course of therapy. Soon (about 24 hours) thereafter, clinical symptoms significantly decreased and could no longer be observed. After continuing the antibiotic and anticoagulant therapy for 4 weeks, a follow-up PET/CT scan was performed. That scan no longer showed abnormal uptake in the previous intravascular localization. Consequently, we suggest that PET/CT is a diagnostic modality feasible to identify and monitor therapy response of intravascular thrombophlebitic foci.

## Abstract (tr)

Nedeni bilinmeyen ateş (NBA) tanı ve tedavi açısından zorluk oluşturmaktadır. 18F-Florodeoksiglukoz pozitron emisyon tomografi-bilgisayarlı tomografi’nin bu klinik durumun ayırıcı tanısındaki rolü günümüzde iyi belirlenmiştir. PET/BT’nin infeksiyöz- inflamatuvar belirtileri olan fakat görünür bir lokalizasyon ve takipte nüks saptanamayan bir hastada sadece NBA etkeni olarak tromboflebit odağını belirlemekle kalmayıp aynı zamanda tedavinin başarısını belirleme ve izlemede etkinliğini gösterdiği bir olguyu sunmaktayız. FDG-PET/BT uygulayarak brakiyosefalik vende tromboflebit belirlendikten sonra, tedavi protokolüne düşük molekül ağırlıklı heparinler eklendi. Çok kısa bir süre (yaklaşık 24 saat) sonra, klinik semptomlar belirgin olarak azaldı ve artık izlenemez oldu. Antibiyotik ve antikoagülan tedavilere 4 hafta devam edildikten sonra, takip PET/BT uygulandı. Bu tetkikte önceki damar içi lokalizasyonda anormal bir tutulum artık izlenmiyordu. Sonuç olarak, PET/BT’nin damar içi tromboflebit odağını belirleme ve tedaviye yanıtı izlemede uygun bir tanı aracı olduğunu düşünmekteyiz.

## INTRODUCTION

Evaluation of fever of unknown origin (FUO) is a well-established indication for 18F-fluorodeoxyglucose positron emission tomography (FDG-PET) / computed tomography (CT) scan as it sometimes might be a challenge for the clinician to determine the cause of a fever after ruling out the most common causes (1,2). Often, despite having gone through the whole diagnostic work-up, including different imaging modalities as well as extensive clinical examinations, it still remains impossible to identify the focus causing the fever. Deep venous thrombosis, sometimes resulting in thrombophlebitis, leads to systemic inflammatory reaction of the body including fever. Identifying the underlying cause of the fever is of crucial importance in the course of optimal therapeutic approach (2). In this setting, FDG-PET was shown to be one of the most reliable diagnostic modalities and therefore of value in the clinical setting of FUO. FDG-PET correlates well with the metabolic activity of a certain lesion such as inflammation (3,4,5). FDG-PET/CT is therefore not only of value in the initial localization of the underlying cause of FUO but might also serve as a surrogate marker for a positive therapy response by showing decreased metabolic (inflammatory) activity.

## CASE REPORT

**Figure 1:**
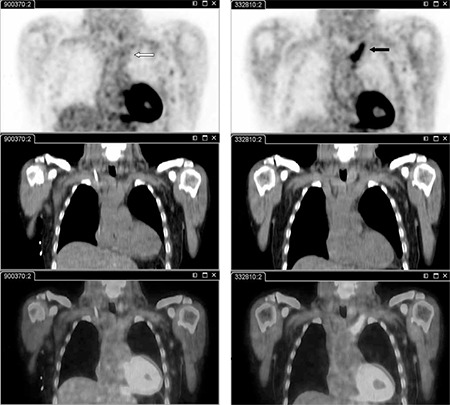
FDG-PET/CT in the diagnosis and treatment evaluation of deep septic thrombophlebitis. Coronal PET, CT and fused PET/CT images (low dose CT used for attenuation correction and localization). Original PET/CT scan (black arrow) and the follow up PET/CT 4 weeks later (white arrow). The activity in the brachiocephalic vein that is seen on the initial PET (black arrow) cannot be found on the follow up PET scan (white arrow).

A 60-year old male patient was admitted to the hospital because of FUO. Additionally, he presented with a generalized malaise, and subtle neurologic symptoms. Blood sample tests were positive for staphylococcus aureus and revealed increased CRP, leucocytes and blood sedimentation. One month before the admission to the hospital, the patient had self extracted an infected toe nail. Besides the positive blood culture, no other examinations including urine culture, repeated echocardiography of the heart, CT of the cerebrum and thorax, and fecal cultures showed any indication for a bacterial focus. He was treated with intravenous and per oral antibiotics (flucoxacillin, clindamycin) during which the fever and local complaints subsided and inflammatory and microbiological parameters in blood normalized. He was discharged but soon again admitted with shivering fever and collapse. Now, a FDG-PET/CT was performed and showed an occlusion in the left brachiocephalic vein which was very intensely PET-positive, attributable to a thrombosis with thrombophlebitis, possibly with associated infection. Additionally, several small foci of increased FDG uptake were seen in the lungs caused by probably infected emboli (Figure 1). A subsequently performed ultrasound examination of the neck revealed a thrombus in the left jugular vein and also in the left supraclavicular vein.

Based on the results of the FDG-PET/CT, low molecular heparin (in a therapeutic dose) was added to the antibiotic therapy. Clinical improvement was observed shortly thereafter. Fever disappeared about 1-2 days after the introduction of low molecular heparin. Additionally, CRP dropped to values of 8 mg/dl, from values between 18 and 40 mg/dl and did not rise again until the end of treatment. Four weeks after the first, a second FDG-PET/CT was performed before patient’s discharge from the hospital. No abnormal FDG uptake could be shown in the intravascular localization and intrathoracic foci. Clinically, all previous foci had disappeared. The patient was than discharged and successfully continued with the treatment of oral antibiotics for another 2 weeks, and anticoagulants for about one year. Clinical follow up examinations during that time course remained negative for a relapse of the infection and/or thrombosis.

**Literature Review and Discussion**

The salient feature of this case report is the use of a FDG-PET/CT scan for the monitoring of the success of treatment of deep septic thrombophlebitis. In our patient, FDG-PET/CT scan was also crucial for the diagnosis of a septic thrombophlebitis in the brachiocephalic vein. Some studies have reported FDG-PET/CT scanning in venous thrombotic disorders. Miceli et al. (1) reported 9 cases of septic deep venous thrombophlebitis localized in vena cava, innominate, subclavian, and internal jugular veins, showing in one case histopathology of an organizing thrombus with neutrophillic cell infiltrate in the vessel wall as the cause for increased FDG uptake. Sopov et al. (3) have made a distinction between non-metabolically active, so-called “bland” thrombi and metabolically active, FDG-positive thrombi as a result of 1. vascular wall inflammation (phlebitis), 2. systemic inflammatory disorder (vasculitis), 3. infection, 4. catheter-related, or 5. tumor tissue thrombus. Although the diagnosis in our patient was not confirmed by histological examination of the thrombus, it is most likely that in our patient central venous thrombosis and -phlebitis was caused by septicemia after septic toe nail extraction and recurrent thrombophlebitis as a result of previous infusion of intravenous antibiotics. In this situation follow-up PET-CT was very valuable to indicate reconvalescence in monitoring successful treatment with oral antibiotics and anticoagulants after previous relapse, and for the prognosis and safe hospital discharge of the patient. Other imaging modalities such as CT and ultrasonography might also be able to detect deep venous thrombosis (6,7), however they can not conclude whether the vascular occlusion is infected or not. In contrast, noninvasive diagnosis of infectious changes is a major advantage of FDG-PET, knowing that leukocytes concentrating in sites of infection, accumulate glucose for their respiratory burst - reaction used for fighting infection (8). This correlates directly with the intensity of FDG accumulation and facilitates the detection of infectious foci. The additional value of combined PET and CT imaging is based on the fact that both anatomic and metabolic information are obtained by the hybrid imaging modality, enabling exact localisation in particular vessels. Furthermore, as in our case whole-body FDG-PET/CT is able to detect potentially multiple active embolic. In the context of fever of unknown origin, such as in our patient FDG-PET/CT performed with high accuracy and a pooled sensitivity and specificity origin of 0.982 and 0.859 respectively (9). In conclusion we suggest that non-invasive therapy evaluation of deep venous thromboflebitis seems possible with FDG-PET/CT and can provide valuable information about additional infectious localisations and success of treatment.

## Figures and Tables

**Figure 2:**
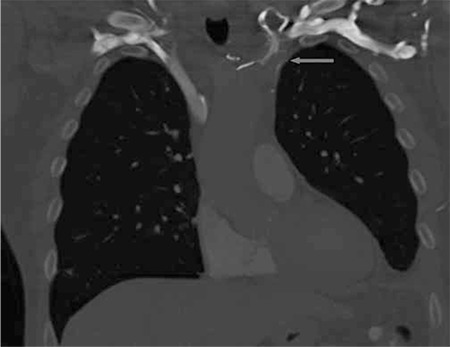
A coronal image reconstruction of a diagnostic CT scan with intravenous iodine contrast, made by the PET/CT. There is a contrast defect (gray arrow) in the proximal left innominate (brachiocepahlic) vein and formation of collateral flow, chages attributable to a thrombus. The latter was consequently confirmed by sonography.
